# Interactions between the amygdala and medial prefrontal cortex as upstream regulators of the hippocampus to reconsolidate and enhance retrieved inhibitory avoidance memory

**DOI:** 10.1186/s13041-021-00753-2

**Published:** 2021-03-02

**Authors:** Hotaka Fukushima, Yue Zhang, Satoshi Kida

**Affiliations:** 1 Department of Bioscience, Faculty of Life Sciences, Tokyo University of Agriculture, 1-1-1, Sakuragaoka, Setagaya-ku, Tokyo, 156-8502 Japan; 2 Graduate School of Agriculture and Life Sciences, The University of Tokyo, 1-1-1 Yayoi, Bunkyo-ku, Tokyo, 113-8657 Japan

**Keywords:** Memory reconsolidation, Amygdala, Medial prefrontal cortex, Hippocampus, Memory retrieval, Memory enhancement

## Abstract

Memory reconsolidation is thought to maintain or enhance an original memory or add new information to the memory. Retrieved inhibitory avoidance (IA) memory is enhanced through memory reconsolidation by activating gene expression in the amygdala, medial prefrontal cortex (mPFC), and hippocampus. However, it remains unclear how these regions interact to reconsolidate/enhance IA memory. Here, we found the interactions between the amygdala and mPFC as upstream regulators of the hippocampus for IA memory reconsolidation. Pharmacological inactivation of the amygdala, mPFC, or hippocampus immediately after IA memory retrieval blocked IA memory enhancement. More importantly, inactivation of the amygdala or mPFC blocked the induction of c-Fos in the amygdala, mPFC, and hippocampus, whereas hippocampal blockade inhibited it only in the hippocampus. These observations suggest interactions between the amygdala and mPFC and they both function as upstream regulators of the hippocampus to reconsolidate IA memory. Our findings suggest circuitry mechanisms underlying IA memory enhancement through reconsolidation between the amygdala, mPFC, and hippocampus.

Memory retrieval is not a passive process, but rather opens memory processes to modify and/or update memory. Retrieved memory becomes labile and is re-stabilized through memory reconsolidation, which requires gene expression activaton [1–9]. Memory reconsolidation is thought to maintain or enhance an original memory or add new information to the memory [6, 8, 10–12]. Previously, we showed that retrieved inhibitory avoidance (IA) memory is enhanced through memory reconsolidation, which requires gene expression in the amygdala, medial prefrontal cortex (mPFC), and hippocampus [9]. Importantly, inhibition of protein synthesis in the amygdala disrupts retrieved IA memory, while this inhibition in the mPFC or hippocampus blocks IA memory enhancement without disruption, suggesting that the amygdala is required for the reconsolidation/enhancement of IA memory, whereas the mPFC and hippocampus are required for its enhancement. These findings suggest that the neural networks between the amygdala, mPFC, and hippocampus are required for IA memory reconsolidation/enhancement, but the amygdala plays distinct roles from the mPFC and hippocampus. However, it remains unknown how these brain regions interact to reconsolidate/enhance IA memory. In this study, we examined the mechanisms underlying IA memory reconsolidation/enhancement by examining the interactions between the amygdala, mPFC, and hippocampus following IA memory retrieval.

We initially examined the effects of inactivation of the amygdala, mPFC (infralimbic and prelimbic regions), or hippocampus on IA memory reconsolidation/enhancement using micro-infusion of the sodium channel blocker lidocaine (LIDO) into these regions (Fig. 1a–c). In the IA task [9], the mice were placed in the light compartment. At 5 s after they entered the dark compartment from the light compartment, an electrical footshock was delivered (Training). The mice were re-exposed to the light compartment at 24 h after Training (Reactivation) and their crossover latency to enter the dark compartment was assessed. The mice were returned to their home cages immediately after they entered the dark compartment without receiving a footshock. Immediately after Reactivation, the mice received a micro-infusion of LIDO or vehicle (VEH) into the amygdala, mPFC, or hippocampus. After 48 h, crossover latency was assessed twice with a 48 h interval (PR-LTM-1 and -2). Two-way repeated analysis of variance (ANOVA) identified significant effects of Drug and Drug vs. Time (Fig. 1a–c; Additional file 1). Consistent with our previous study [9], post hoc Bonferroni analysis revealed that the VEH groups showed significantly increased crossover latency at PR-LTM-1 compared to Reactivation, indicating that IA memory retrieval in the light compartment enhanced the memory (Fig. 1a–c). In contrast, the LIDO groups showed comparable and significantly less crossover latency at PR-LTM-1 compared to Reactivation and the VEH groups, respectively, indicating that inactivation of the amygdala, mPFC or hippocampus immediately after Reactivation blocks IA memory enhancement (Fig. 1a–c). Importantly, the LIDO groups showed significantly more and comparable crossover latency at PR-LTM-2 compared to PR-LTM-1 and the VEH groups at PR-LTM-1, respectively, suggesting that the LIDO groups showed IA memory enhancement at PR-LTM-2 in the absence of LIDO micro-infusion at PR-LTM-1. Collectively, our observations indicated that inactivation of these regions by LIDO blocked retrieval-induced IA memory enhancement.

**Fig. 1:**
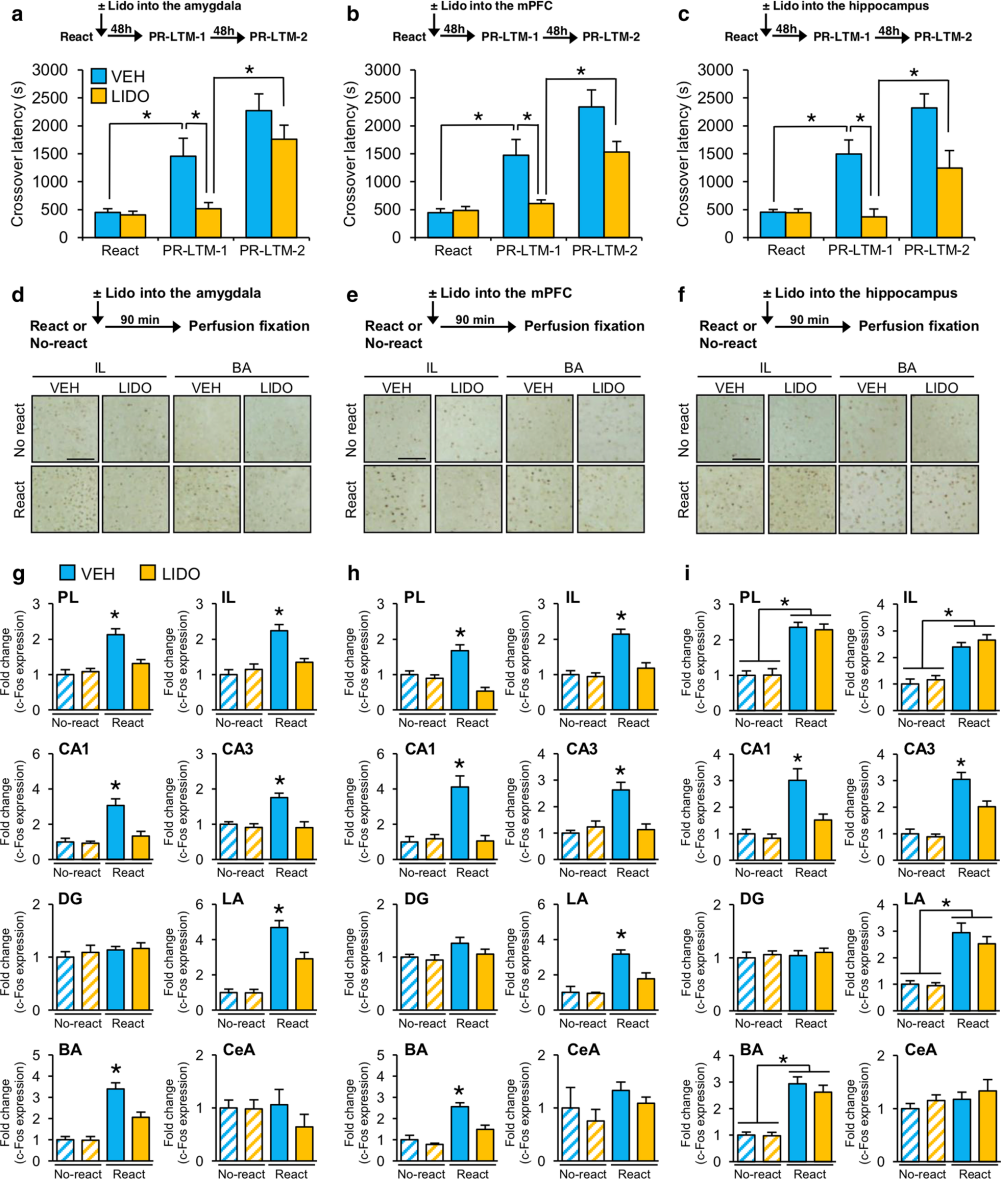
Effects of inactivation of the amygdala, mPFC, or hippocampus on the induction of c-Fos expression.** a**–**c** Micro-infusion of LIDO into the amygdala (**a** VEH, n = 8, LIDO, n = 11), mPFC (**b** VEH, n = 9, LIDO, n = 9) or hippocampus (**c** VEH, n = 10, LIDO, n = 9). *p < 0.05; two-way repeated ANOVA followed by the post hoc Bonferroni test. **d**–**f** Experimental time-course and representative immunohistochemical staining of c-Fos-positive cells in the BA and IL from the indicated group. Scale bar, 100 μm. **g**–**i** c-Fos expression in the PL and IL of the mPFC, CA1, CA3, and DG regions of the hippocampus, and LA, BA, and CeA regions of the amygdala 90 min after the micro-infusion of LIDO or VEH into the amygdala (**g**), mPFC (**h**) or hippocampus (**i**). n = 7–15 for each group. *p < 0.05; two-way ANOVA followed by the post hoc Bonferroni test. *ANOVA* analysis of variance, *BA* basolateral, *CeA* central, *DG* dentate gyrus, *IL* infralimbic, *LA* lateral, *LIDO* lidocaine, *No-react* No reactivation, *PR-LTM-1* post-reactivation long-term memory test-1, *PR-LTM-2* post-reactivation long-term memory test-2, *PL* prelimbic, *React* Reactivation, *VEH* vehicle. Error bars, standard error of the mean. The results of the statistical analyses are presented in Additional file 1

We next examined the effects of inactivation of the amygdala, mPFC, or hippocampus by LIDO micro-infusion on the induction of c-Fos expression in the other regions following Reactivation using immunohistochemistry [9]. We performed a similar experiment as in Fig. 1a–c, except that c-Fos expression was assessed at 90 min after Reactivation [Reactivation (React) groups]. The no-reactivation (No-react) groups were trained, but not re-exposed to the light compartment. The number of c-Fos-positive cells was counted in the amygdala, mPFC, and hippocampus [9]. Importantly, inactivation of the amygdala or mPFC in the React groups inhibited the induction of c-Fos expression in the other brain regions, although this inactivation in the No-react groups had no effect on c-Fos expression in the other brain regions (Fig. 1g, h). Two-way ANOVA identified a significant Drug vs. Reactivation (Re-exposure or No re-exposure) interaction in the hippocampus (CA1 and CA3), amygdala (lateral and basolateral regions), and mPFC (prelimbic and infralimbic regions) (Additional file 1). The React groups infused with VEH into the amygdala or mPFC showed significant c-Fos induction in the amygdala, mPFC and hippocampus compared to the No-react groups. In contrast, the React groups infused with LIDO into the amygdala or mPFC showed significantly lower levels of c-Fos expression in the brain regions compared to the React-VEH groups. These observations indicate that inactivation of the mPFC or amygdala inhibits c-Fos induction in the amygdala, mPFC, and hippocampus.

In contrast, inactivation of the hippocampus inhibited c-Fos induction only in the CA1 and CA3 regions of the hippocampus, without affecting c-Fos expression in the amygdala and mPFC (Fig. 1i). Two-way ANOVA identified a significant Drug vs. Reactivation interaction in the hippocampus (CA1 and CA3), but not amygdala and mPFC (Additional file 1). The React group infused with VEH into the hippocampus showed significant c-Fos induction in the amygdala, mPFC and hippocampus compared to the No-react groups. In contrast, the React-LIDO group showed significantly lower levels of c-Fos expression in the hippocampus, but showed comparable expression in the amygdala and mPFC, compared to the React-VEH group. These observations indicate that inactivation of the hippocampus failed to affect c-Fos expression in the other two regions. Collectively, our data suggest that the amygdala and mPFC interact with each other and function as upstream regulators of the hippocampus.

We previously showed that the amygdala is required for IA memory reconsolidation, while the mPFC and hippocampus are required for its enhancement, but not reconsolidation [9], suggesting that the amygdala plays a central role in IA memory reconsolidation/enhancement after retrieval. However, the current study showed that the amygdala and mPFC contribute equally to IA memory reconsolidation/enhancement by interacting with each other.

Our observations suggest that the hippocampus contributes to IA memory enhancement after retrieval as a downstream regulator of the amygdala and mPFC. Although the hippocampus receives direct input from the amygdala [13], it remains unknown how the mPFC regulates the hippocampus, since the hippocampus does not receive direct input from the mPFC. It is possible that the hippocampus is regulated indirectly by the mPFC via the amygdala. It is important to identify the neural circuits that mediate input from the mPFC to the hippocampus.

In summary, our observations suggest that the amygdala and mPFC interact with each other and function as upstream regulators of the hippocampus to reconsolidate/enhance retrieved IA memory and that neural networks among these regions regulates IA memory reconsolidation/enhancement.

## Supplementary Information

**Additional file 1.** Containing detailed material and methods, statistics and sample sizes, and cannulation tip placements.

## Data Availability

All data generated or analyzed during this study are included in this published article and its additional information file.

## Abbreviations

IA: Inhibitory avoidance
mPFC: Medial prefrontal cortex
LIDO: Lidocaine
VEH: Vehicle
PR-LTM-1: Post-reactivation long-term memory test-1
PR-LTM-2: Post-reactivation long-term memory test-2
React: Reactivation
No-react: No-reactivation
