# Supplementary material for: Interactions between the amygdala and medial prefrontal cortex as upstream regulators of the hippocampus to reconsolidate and enhance retrieved inhibitory avoidance memory
Source: Mol Brain. 2021 Mar 2;14:44. doi: 10.1186/s13041-021-00753-2 (PMC7923328; doi:10.1186/s13041-021-00753-2)
Supplement: Supplementary file 1 — Additional file 1. Containing detailed material and methods, statistics and sample sizes, and cannulation tip placements. [file 13041_2021_753_MOESM1_ESM.docx]

**Additional file 1**

**Materials and Methods**

**Mice.**

All experiments were conducted according to the *Guide for the Care* and *Use of Laboratory Animals* (Japan Neuroscience Society and Tokyo University of Agriculture). All animal experiments performed in this study were approved by the Animal Care and Use Committee of Tokyo University of Agriculture (authorization #100558). All surgical procedures were performed under Nembutal anesthesia and every effort was made to minimize suffering. Male C57BL/6N mice were obtained from Charles River (Yokohama, Japan). The mice were housed in cages of 5 or 6, maintained on a 12-h light/dark cycle, and allowed access to food and water *ad libitum*. The mice were at least 8 weeks of age when tested. Testing was performed during the light phase of the cycle. All experiments were conducted blind to the treatment condition of the mice.

**Drug**

The sodium channel blocker lidocaine (LIDO, 4%; Sigma-Aldrich, St. Louis, MO, USA) was dissolved in phosphate-buffered saline (PBS)^1^.

**IA test**

The step-through IA apparatus (OHARA Pharmaceutical, Tokyo, Japan) consisted of a box with separate light and dark compartments (both 15.5 × 12.5 × 11.5 cm). The light compartment was illuminated by a fluorescent light (2500 lux)^2-5^. Before the commencement of IA training, the mice were handled individually for 2 min each day for 1 week. During the training sessions, each mouse was allowed to habituate to the light compartment for 30 s, and the guillotine door was raised to allow access to the dark compartment. Latency to enter the dark compartment was considered as a measure of acquisition. As soon as the mouse had entered the dark compartment, the guillotine door was closed. After 5 s, a footshock (0.2 mA) was delivered for 2 s (Training). At 24 h after Training, the mouse was placed back in the light compartment until it entered the dark compartment without a footshock (Reactivation). Memory was assessed twice at an interval of 48 h (PR-LTM-1 and -2) as the crossover latency for the mouse to enter the dark compartment when replaced in the light compartment, as in Reactivation. The Reactivation session was terminated at 3000 s.

For the first experiment (LIDO micro-infusion), we examined the effects of inactivation of the amygdala, mPFC, or hippocampus on the reconsolidation/enhancement of IA memory. The mice were trained as described above, and at 24 h later, they were placed back in the light compartment (Reactivation). The mice were micro-infused with LIDO (20 µg) or vehicle (VEH) into the amygdala, mPFC or hippocampus immediately after Reactivation. At 48 h after Reactivation, the mice were once again placed in the light compartment and crossover latency was assessed twice at an interval of 48 h (PR-LTM-1 and -2). Micro-infusions into the hippocampus and mPFC (0.5 µL) were performed at a rate of 0.25 μL/min. Micro-infusions into the amygdala (0.2 µL) were performed at a rate of 0.1 μL/min. The injection cannula was left in place for 2 min after micro-infusion and the mice were then returned to their home cages.

For the second experiment (c-Fos immunohistochemistry), we examined the effects of inactivation of the amygdala, mPFC, or hippocampus by LIDO micro-infusion on the induction of c-Fos expression in these brain regions following Reactivation. The mice were trained and micro-infused with LIDO or VEH into the amygdala, mPFC or hippocampus immediately after Reactivation as described above [Reactivation (React) groups]. No-reactivation (No-react) groups were trained and at 24 h later they were micro-infused with LIDO or VEH, but not re-exposed to the light compartment. The mice were anesthetized with Nembutal (750 mg/kg, i.p.) at 90 min after Reactivation.

**Immunohistochemistry**

Immunohistochemistry was performed as described previously^3-8^. After anesthetization, the mice were perfused with 4% paraformaldehyde. The brains were removed, fixed overnight, transferred to 30% sucrose, and stored at 4°C. Coronal sections (30 μm) were cut in a cryostat.

For c-Fos staining, the sections were washed and preincubated in 3% H_2_O_2_ in methanol for 1 h, followed by incubation in a blocking solution (PBS plus 1% goat serum albumin, 1 mg/mL bovine serum albumin, and 0.05% Triton X-100) for 3 h. Consecutive sections were incubated with a polyclonal rabbit for anti-c-Fos antibody (Ab-5, 1:5000, RRID: AB_2106755; Millipore, Bedford, MA, USA) in the blocking solution overnight. Subsequently, the sections were washed with PBS and incubated for 3 h at room temperature with biotinylated goat anti-rabbit IgG (SAB-PO Kit; Nichirei Biosciences, Tokyo, Japan), followed by incubation for1 h at room temperature in streptavidin-biotin-peroxidase complex (SAB-PO Kit).

**Quantification of c-Fos positive cells**

Quantification was performed as described previously^3-10^. Structures were defined anatomically according to the atlas of Franklin and Paxinos^11^. All immunoreactive neurons were counted by an experimenter blind to the treatment condition. Quantification of c-Fos-positive cells in sections (100 × 100 μm) of the mPFC (bregma between 2.10 and 1.98 mm), amygdala (bregma between -1.22 and -1.34 mm), and dorsal hippocampus (bregma between -1.46 and -1.82 mm) was performed using computerized image analyses, as described previously^3-10,12^ (WinROOF version 5.6 software; Mitani Corporation, Fukui, Japan). Immunoreactive cells were counted bilaterally with a fixed sample window across at least 3 sections. The expression of c-Fos in each group is expressed as the ratio of the No-react VEH (Fig. 1 G, H and I) group to the other groups.

**Surgery for drug micro-infusion**

Surgery was performed as described previously^3-10,12-14^. Under Nembutal anesthesia and using standard stereotaxic procedures, a stainless-steel guide cannula (22 G) was implanted into the mPFC (2.7 mm, ±0 mm, -1.6 mm), amygdala (-1.3 mm, ±3.3 mm, -4.4 mm), or dorsal hippocampus (-1.8 mm, ±1.8 mm, -1.9 mm). Stereotaxic coordinates for mPFC, dorsal hippocampus, or amygdala placement were based on the atlas of Franklin and Paxinos^11^. The mice were allowed to recover for at least 1 week after surgery. After that, they were handled for 1 week before the commencement of training. Only mice with a cannulation tip within the boundaries of the mPFC, dorsal hippocampus, or amygdala were included in the data analyses. Cannulation tip placements are shown in Additional file 1.

**Data analysis**

Two-way repeated and two-way ANOVA followed by *post hoc* Bonferroni’s comparisons were used to analyze the effects of drug, time, and reactivation. All values in the text and figure legends are means ± standard error of the mean.

**Reference**

1. Frankland PW, Bontempi B, Talton LE, Kaczmarek L, Silva AJ. The involvement of the anterior cingulated cortex in remote contextual fear memory. Science. 2004; 304 (5672), 881–3.
2. Fukushima H, Maeda R, Suzuki R, Suzuki A, Nomoto M, Toyoda H, Wu LJ, Xu H, Zhao MG, Ueda K, Kitamoto A, Mamiya N, Yoshida T, Homma S, Masushige S, Zhuo M, Kida S. Upregulation of calcium/calmodulin-dependent protein kinase IV improves memory formation and rescues memory loss with aging. J Neurosci. 2008; 28 (40), 9910-9.
3. Fukushima H, Zhang Y, Archbold G, Ishikawa R, Nader K, Kida S. Enhancement of fear memory by retrieval through reconsolidation. eLife. 2014; 3, e02736.
4. Ishikawa R, Fukushima H, Frankland PW, Kida S. Hippocampal neurogenesis enhancers promote forgetting of remote fear memory after hippocampal reactivation by retrieval. eLife. 2016; 5, e17464.
5. Zhang Y, Fukushima H, Kida S. Induction and requirement of gene expression in the anterior cingulate cortex and medial prefrontal cortex for the consolidation of inhibitory avoidance memory. Mol Brain. 2011; 4, 4.
6. Mamiya N, Fukushima H, Suzuki A, Matsuyama Z, Homma S, Frankland PW, Kida S. Brain region-specific gene expression activation required for reconsolidation and extinction of contextual fear memory. J Neurosci. 2009; 29 (2), 402–13.
7. Hasegawa S, Fukushima H, Hosoda H, Serita T, Ishikawa R, Rokukawa T, Kawahara-Miki R, Zhang Y, Ohta M, Okada S, Tanimizu T, Josselyn SA, Frankland PW, Kida S. Hippocampal clock regulates memory retrieval via Dopamine and PKA-induced GluA1 phosphorylation. Nat Commun. 2019; 10 (1), 5766.
8. Suzuki A, Fukushima H, Mukawa T, Toyoda H, Wu LJ, Zhao MG, Xu H, Shang Y, Endoh K, Iwamoto T, Mamiya N, Okano E, Hasegawa S, Mercaldo V, Zhang Y, Maeda R, Ohta M, Josselyn SA, Zhuo M, Kida S. Upregulation of CREB-mediated transcription enhances both short- and long-term memory. J Neurosci. 2011; 31 (24), 8786-802.
9. Frankland PW, Ding HK, Takahashi E, Suzuki A, Kida S, Silva AJ. Stability of recent and remote contextual fear memory. Learn Mem. 2006; 13 (4), 451-7.
10. Suzuki A, Mukawa T, Tsukagoshi A, Frankland PW, Kida S. 2008. Activation of LVGCCs and CB1 receptors required for destabilization of reactivated contextual fear memories. Learn Mem. 2008; 15 (6), 426–33.
11. Franklin KB, Paxinos G. The mouse brain in stereotaxic coordinates. San Diego: Elsevier Academic. 1997.
12. Inaba H, Tsukagoshi A, Kida S. PARP-1 activity is required for the reconsolidation and extinction of contextual fear memory. Molecular Brain. 2015;8 (1), 63.
13. Kim R, Moki R, Kida S. Molecular mechanisms for the destabilization and restabilization of reactivated spatial memory in the Morris water maze. Molecular Brain. 2011; 4, 9.
14. Nomoto M, Takeda Y, Uchida S, Mitsuda K, Enomoto H, Saito K, Choi T, Watabe AM, Kobayashi S, Masushige S, Manabe T, Kida S. Dysfunction of the RAR/RXR signaling pathway in the forebrain impairs hippocampal memory and synaptic plasticity. Mol Brain. 2012; 5, 8.

**Statistics and Sample Sizes.**

**Cannulation tip placements**
